# CAR-T cell therapy embarks on autoimmune disease

**DOI:** 10.1038/s41409-024-02429-6

**Published:** 2024-10-08

**Authors:** Alexandros Rampotas, Johanna Richter, David Isenberg, Claire Roddie

**Affiliations:** 1 University College London Hospital NHS Foundation Trust, London, UK; 2 Department for Stem Cell Transplantation, University Medical Center Hamburg-Eppendorf, Hamburg, Germany; 3 Centre for Ageing, Rheumatology and Regenerative Medicine, Division of Medicine, University College London, London, UK

**Keywords:** Immunotherapy, Immunological disorders

## Introduction

Chimeric antigen receptor T cell therapy targeting the B-cell antigen CD19 (CD19CAR-T) has shown remarkable effectiveness against leukaemia/lymphoma following treatment failure with the B-cell targeting monoclonal antibodies (mAbs) Rituximab and Obinatuzumab in combination with chemotherapy [1, 2]. B-cell aplasia is a ubiquitous off-tumour-on-target phenomenon of CD19CAR-T [3].

In autoimmunity where the pathology is often driven by autoreactive B-cells, notably in patients with systemic lupus erythematosus (SLE), B-cell targeting mAbs can be highly effective [4]. However, many SLE patients become refractory or relapse following mAb therapy for SLE [5] which is most likely due to incomplete B-cell eradication from tissue-resident compartments [6]. Evidence in support of this explanation comes from studies showing ongoing memory B-cell persistence despite depletion of circulating B-cells following mAb therapy for SLE [7, 8].

The capacity of CD19CAR-T therapy to achieve tissue clearance of autoreactive B-cells is an attractive prospect for patients with autoimmune conditions in whom mAb therapy fails. From leukaemia/lymphoma experience CAR-T therapy is highly effective in patients when prior B-cell targeting mAb therapy has failed. Furthermore, the pharmacokinetics of CAR-T therapy is distinct from that of mAbs, with evidence of active CAR-T trafficking and biodistribution into even the most immunologically challenging and remote areas, e.g., the central nervous system and skin [9], amongst others [10]. Lastly, CAR-T treatment frequently leads to profound and complete B-cell depletion, albeit with B-cell recovery observed in most patients by around 12 months post-CAR-T infusion [3].

Whilst to date CAR-T therapy has only been tested in a limited number of patients with autoimmune disease (Table 1), it has shown highly promising results and excitingly, may represent a paradigm shift in the management of these challenging conditions.

**Table 1:** Current trials for CAR-T cell therapy in autoimmune diseases.

Target	Conditions	NTC	Phase	Sponsor
CD19	Severe, refractory systemic lupus erythematosus	NCT05869955	Phase 1	Bristol-Myers Squibb
CD19	Systemic Lupus Erythematosus	NCT05765006	Phase 1	Shanghai Ming Ju Biotechnology Co., Ltd.
CD19	Systemic Lupus Erythematosus	NCT03030976	Phase 1	Shanghai GeneChem Co., Ltd.
CD19	Refractory Systemic Lupus Erythematosus	NCT06189157	Phase 1/2	Miltenyi Biomedicine GmbH
CD19	Refractory Lupus nephritis	NCT05938725	Phase 1	Kyverna Therapeutics
CD19	Systemic lupus erythematosuslupus nephritis	NCT05798117	Phase 1/2	Novartis Pharmaceuticals
CD19	Systemic Lupus Erythematosus	NCT06121297	Phase 1/2	Cabaletta Bio
CD19	Refractory/Moderate-to-severe Systemic Lupus Erythematosus	NCT06106906	Phase 1/2	Wuhan Union Hospital, China
CD19/BCMA	Relapsed/refractory systemic lupus erythematosus	NCT05474885	Phase 1	iCell Gene Therapeutics
CD19/BCMA	Refractory systemic lupus erythematosus	NCT05846347	Phase 1	Zhejiang University
CD19/CD20	Refractory Systemic Lupus Erythematosus	NCT06153095	Phase 1/2	ImmPACT Bio
CD19	Idiopathic Inflammatory Myopathy Dermatomyositis Anti-Synthetase-Syndrome Immune-Mediated Necrotizing Myopathy	NCT06154252	Phase 1/2	Cabaletta Bio
CD19	Relapsing/Progressive Forms of Multiple Sclerosis	NCT06220201	Phase 1	Juno Therapeutics, Inc., a Bristol-Myers Squibb Company
CD19	Non-relapsing and Progressive Forms of Multiple Sclerosis	NCT06138132	Phase 1	Stanford University
CD19	Refractory myasthenia gravis	NCT05828225	Phase 1	Zhejiang University
CD19	Refractory Generalized Myasthenia Gravis	NCT06193889	Phase 2	Kyverna Therapeutics
BCMA	Generalised myasthenia gravis	NCT04146051	Phase 2	Cartesian Therapeutics

Selected clinical trials for different CAR-T cell constructs in distinct autoimmune diseases.

### Rheumatology

Many rheumatological diseases are driven by the formation of autoantibodies produced by dysfunctional B-cell clones [11]. Georg Schett and his colleagues at Erlangen University have provided pivotal proof-of-concept evidence for CD19CAR-T in three autoimmune rheumatic diseases (ARD) by establishing a local CAR-T manufacture protocol in collaboration with Miltenyi Biotech [12] and treating mAb-refractory rheumatology patients on an investigator-initiated study [13]. Thus they reported their CD19CAR-T experience in eight patients with SLE, three with idiopathic inflammatory myositis, and four with systemic sclerosis [13]. In these patients, CD19CAR-T was found to be safe, with no grade 3 cytokine release syndrome (CRS) or neurotoxicity, and only one case of pneumonia necessitating hospitalization, while three patients required Tocilizumab for low-grade CRS. Incredibly, all patients showed significant improvement in disease-specific activity scores accompanying a rapid loss of B-cells, without a requirement for further immunosuppressive therapy at a median follow-up of 15 months and in one case now for over three years. In those with SLE, a reduction in dsDNA antibodies and normalisation of complement C3 and C4 levels was observed. The median duration of B-cell aplasia was only 112 days (range 65–159) suggesting that even transient B-cell aplasia can serve a therapeutic role in antibody-mediated autoimmune disease. Further, CD19CAR-T treated patients with restored B-cell function were able to generate antibody responses successfully to vaccinations of common pathogens (measles, rubella, mumps, varicella zoster virus and hepatitis B, tetanus, diphtheria and pneumococci) at 3 months post-treatment, illustrative of numeric and functional B-cell recovery [14].

Beyond CD19, other targets of interest in rheumatological disease include B-cell maturation antigen (BCMA), a highly expressed cell surface protein on plasma cells. Briefly, in a clinical trial of BCMA-CD19 compound CAR-T cells (cCAR) for SLE and lupus nephritis (LN), cCAR was well-tolerated, with only mild cytokine release syndrome observed [15].12/13 cCAR-treated patients achieved a reduction in SLE Disease Activity Index 2000 (SLEDAI-2K) score from 10.6 at baseline to 2.7 3 months post-cCAR, and 9/13 patients achieved complete symptom and medication-free remission, with significant improvements in renal function in 10/13 patients. Further data on the role of BCMA-CAR in rheumatological disease are eagerly awaited.

### Neurology

Separately, the use of CD19CAR-T is being explored as a mode of immune modulation in inflammatory and autoantibody-driven neurological pathologies such as myasthenia gravis (MG) and multiple sclerosis (MS) that are refractory to B-cell depleting/suppressing mAbs. Ongoing clinical CAR-T trials in neurology are listed in Table 1. An illustrative case of CD19CAR-T for MG reported a 70% reduction in pathogenic anti-AchR antibodies at day 62 post-CAR treatment and a full restoration of function ongoing at month 2 post-CAR-T in a patient who had failed prior proteasome inhibitor, mycophenolate mofetil and immunoglobulin infusion therapy, and in the absence of severe immuno- or neurotoxicity [16]. Additionally, initial results from a BCMA targeting mRNA CAR-T cell therapy phase Ib/IIa trial (NCT04146051) for patients with generalised MG (*N* = 16) showed that CAR-T therapy was well-tolerated with no dose-limiting toxicities, cytokine release syndrome, or neurotoxicity. Clinically, the treatment led to significant improvements in myasthenia gravis severity scales, with mean reductions across all MG-related scores (14 points reduction in Myasthenia Gravis Composite score) by week 12, with these effects persisting so far up to a median of 5 months (2–9 months) [17]. Furthermore, a case of refractory anti-synthetase syndrome exhibited excellent response to CD19CAR-T therapy with minimal to no toxicity after 200 days of follow-up [18]. Currently, a phase 1 trial is in progress in the United States of America to evaluate the use of CD19CAR-T therapy for multiple sclerosis [19] with initial case reports showing safety and preliminary efficacy for CD19CAR-T cells in patients with progressive MS [20]. Whilst this represents early data, so far CAR-T for neurological conditions does not appear to present a higher risk of neurotoxicity despite the location of the pathology.

### Lessons to be learned

CAR-T therapy heralds a new era in autoimmune disease management, offering a targeted and potent immunomodulatory approach that holds promise for inducing durable remissions through an immune system ‘reset’. In contrast to malignant disease where CAR-T persistence can be durable, the short-duration CAR-T engraftment observed thus far in the autoimmunity setting may also limit the risks of longer-term CAR-T-associated immune dysfunction and infection susceptibility. Additionally, an mRNA CAR-T product that should have limited persistence has also shown remarkable responses even at longer follow-ups [18]. This emerging difference illustrates that the mechanisms underlying CAR-T persistence are complex [21]. With the important caveat that the numbers of patients treated thus far are very small, it is encouraging thus far that CAR-T for autoimmune conditions appears to be safe, with little/no immunotoxicity perhaps because lower doses of CAR-T cells were used compared with malignant disease as well as an overall lower antigen burden.

The clinical and serological improvement seen in diverse ARD and neurological autoimmune diseases has been genuinely dramatic but as yet it is not known how long the benefit will last. It is possible that say 5 years after the treatment with CAR-T these diseases may all relapse. Given the short-duration CAR-T persistence observed thus far in autoimmunity, it will be interesting to evaluate the role of repeated CAR-T dosing. Whilst repeat dosing has yielded limited clinical benefit in the B-cell malignancy setting [22], it is possible that a different paradigm can be applied in the autoimmunity setting.

## The future

B-cell targeting CAR-T has the potential to ‘reset’ the immune system by clearing autoreactive B-cells, selectively permitting normal naïve B-cell reconstitution. The restoration of normal B-cell function raises important questions about the potential for autoimmune disease relapse over longer follow-up periods. If relapses are found to be extremely rare, this would suggest that these autoimmune diseases may result from specific triggers that mislead the immune system into self-attack, rather than from a genetic and hormonal predisposition that simply requires a trigger to become active. Another hypothesis, if long-term remissions are confirmed, is that CAR-T cells could materially change the B-cell population and remove a B-cell clone that was created following multiple antigen stimulations and hence it is challenging to re-emerge.

Despite promising results in SLE, scleroderma and myositis, there remain a number of challenges for the field. Briefly, the exact role for B-cell depletion and CD19CAR-T in some other autoimmune diseases remains controversial. In some diseases, autoantibodies reflect a more T cell-driven autoimmune response [23], and in others, autoantibodies are secreted by plasmablasts/plasma cells which do not express CD19. The role of lymphodepletion with fludarabine and cyclophosphamide should be better clarified as some of the responses may be driven by the initial T cell suppression and prove to be only transient. Identifying the patients, within each disease entity who stand to gain the most from such treatments is essential, particularly because administering this therapy at advanced stages of the disease, where irreversible organ damage has already occurred, may limit the possible benefit. A key inquiry remains as to whether CAR-T cell therapy can be extended to treat other autoimmune conditions such as rheumatoid arthritis, Sjögren’s syndrome, and type 1 diabetes. Timing is crucial in diseases like type 1 diabetes where complete elimination of insulin-producing pancreatic beta cells may not be reversed even after the elimination of autoreactive T/B-cells. As for rheumatoid arthritis, the high costs associated with these therapies may limit their use to only the most severe cases, given the prevalence of the condition and the widespread availability of effective monoclonal therapies. Utilization of allogeneic CAR-T cells or NK CARs which could potentially be used off the shelf may provide a solution to the challenges of cost and ease of access. One of the main obstacles in their use in the malignant setting is the lack of persistence as eventually they are rejected by the host. Given that long-term persistence of the cells is not desirable on autoimmune diseases, to allow the immune system to recover fully, these therapeutics may be proven to be more suited on this setting with trial results eagerly awaited [24]. However, these therapies will require significant T cell suppression as conditioning to allow these cells to proliferate and work, unless genome editing strategies are employed to delete HLA-class molecules. Such strategy was used on NCT05859997 trial where allogeneic CD19 CRISPR edited CAR-T cells were used. Initial results from three cases of myositis (*N* = 1) and systemic sclerosis (*N* = 2) have shown promising outcomes with complete remissions and no significant side effects for all three cases after 6 months of follow-up [25].

### Conclusion

The current preliminary results of CAR-T use for antibody-mediated autoimmune diseases are very appealing. Nevertheless, significant efforts are needed to expand CAR-T therapy to other autoimmune diseases. CARs bearing a humanised CD19 binder, or bispecific CARs also targeting BCMA could further improve their efficacy in autoimmune disease. Improvements in manufacturing and turnaround time could even make them a viable therapy for diseases such as type 1 diabetes. Concerns in relation to the high initial costs associated with CAR-T should be balanced with the lifelong financial burden of these chronic and often debilitating diseases. Better constructs and improvements in manufacturing could mean that CAR-T products could in the future, dominate the treatment options for autoimmune disease.
